# 13 Thromboelastographic Coagulation Assessment in Burn Patients Receiving Plasma Inclusive Resuscitation

**DOI:** 10.1093/jbcr/irae036.013

**Published:** 2024-04-17

**Authors:** Shane Mathew, Tuan D Le, Anthony Pusateri, Shawn Tejiram, Taryn E Travis, Bonnie C Carney, Melissa M McLawhorn, Lauren T Moffatt, Jeffrey W Shupp

**Affiliations:** MedStar Washington Hospital Center, Washington, District of Columbia; Naval Medical Research Unit San Antonio, JBSA-Fort Sam Houston, San Antonio, Texas; Georgetown University School of Medicine, Washington, DC; MedStar Health Research Institute, Washington, District of Columbia; Firefighters’ Burn and Surgical Research Laboratory, Washington, DC; MedStar Health; MedStar Washington Hospital Center, Washington, District of Columbia; Naval Medical Research Unit San Antonio, JBSA-Fort Sam Houston, San Antonio, Texas; Georgetown University School of Medicine, Washington, DC; MedStar Health Research Institute, Washington, District of Columbia; Firefighters’ Burn and Surgical Research Laboratory, Washington, DC; MedStar Health; MedStar Washington Hospital Center, Washington, District of Columbia; Naval Medical Research Unit San Antonio, JBSA-Fort Sam Houston, San Antonio, Texas; Georgetown University School of Medicine, Washington, DC; MedStar Health Research Institute, Washington, District of Columbia; Firefighters’ Burn and Surgical Research Laboratory, Washington, DC; MedStar Health; MedStar Washington Hospital Center, Washington, District of Columbia; Naval Medical Research Unit San Antonio, JBSA-Fort Sam Houston, San Antonio, Texas; Georgetown University School of Medicine, Washington, DC; MedStar Health Research Institute, Washington, District of Columbia; Firefighters’ Burn and Surgical Research Laboratory, Washington, DC; MedStar Health; MedStar Washington Hospital Center, Washington, District of Columbia; Naval Medical Research Unit San Antonio, JBSA-Fort Sam Houston, San Antonio, Texas; Georgetown University School of Medicine, Washington, DC; MedStar Health Research Institute, Washington, District of Columbia; Firefighters’ Burn and Surgical Research Laboratory, Washington, DC; MedStar Health; MedStar Washington Hospital Center, Washington, District of Columbia; Naval Medical Research Unit San Antonio, JBSA-Fort Sam Houston, San Antonio, Texas; Georgetown University School of Medicine, Washington, DC; MedStar Health Research Institute, Washington, District of Columbia; Firefighters’ Burn and Surgical Research Laboratory, Washington, DC; MedStar Health; MedStar Washington Hospital Center, Washington, District of Columbia; Naval Medical Research Unit San Antonio, JBSA-Fort Sam Houston, San Antonio, Texas; Georgetown University School of Medicine, Washington, DC; MedStar Health Research Institute, Washington, District of Columbia; Firefighters’ Burn and Surgical Research Laboratory, Washington, DC; MedStar Health; MedStar Washington Hospital Center, Washington, District of Columbia; Naval Medical Research Unit San Antonio, JBSA-Fort Sam Houston, San Antonio, Texas; Georgetown University School of Medicine, Washington, DC; MedStar Health Research Institute, Washington, District of Columbia; Firefighters’ Burn and Surgical Research Laboratory, Washington, DC; MedStar Health; MedStar Washington Hospital Center, Washington, District of Columbia; Naval Medical Research Unit San Antonio, JBSA-Fort Sam Houston, San Antonio, Texas; Georgetown University School of Medicine, Washington, DC; MedStar Health Research Institute, Washington, District of Columbia; Firefighters’ Burn and Surgical Research Laboratory, Washington, DC; MedStar Health

## Abstract

**Introduction:**

As resuscitative strategies in burn patients are re-evaluated, a consensus has not been reached on the role for colloids, as lactated ringers remain the fluid of choice with variable use of colloids. Colloids, such as fresh frozen plasma (FFP), may be effective at maintaining oncotic pressure, resupplying coagulation factors, mitigating coagulopathy, and treating the endothelium. This work aims to characterize the coagulation profile of burn patients treated with plasma inclusive resuscitation (PIR) using rapid thromboelastography (rTEG) by comparing it to burn patients primarily resuscitated with crystalloids alone.

**Methods:**

Patients admitted to a regional burn center who had greater than 20% total body surface area (TBSA) burns that received PIR were included in the study. Patients admitted with burn injuries and resuscitated primarily with lactated ringers were included in the comparison group. Demographic data and blood samples were collected at 4 time points: baseline, before FFP, immediately following the initial unit of FFP, and following the completion of consecutive units of FFP. Samples collected from patients that received LR only were approximated to the FFP group time points. Coagulation parameters were measured using rTEG in blood samples. Descriptive analysis was used to compare demographic and burn injury characteristics at admission. A mixed effects model was used to compare the two groups through the observation period overall and at each time point.

**Results:**

There were 35 patients in the FFP group and 15 patients in the comparison group. The groups were primarily male (74.3% vs 73.3%), and there was no difference in demographics, TBSA (34.0% (27.0-48.5) vs 33.0% (26.5-46.5, p=0.82)), Baux score, inhalation injury, and mortality rate (28.6% vs 26.7%, p=0.99). Major coagulation markers of rTEG (k, α-angle, MA, and ly30) were not different between these groups and were within normal ranges. The R time in the crystalloid group was greater than the normal range at all 4 time points. There were significant differences in R time at pre-FFP (30.0(25.0-32.5) secs vs 48.0 (36.0-54.0) secs, p< 0.001) and after all units (27.5(20.9-33.8) secs vs 48.0(48.0-54.0) secs, p< 0.0001) when compared to the FFP group where R time was in the normal range.

**Conclusions:**

Plasma inclusive burn resuscitation did not result in hypercoagulability or changes in lysis when compared to patients primarily resuscitated with crystalloids. The elevated R time in the crystalloid group, which was not seen in the FFP group, may be due to the lack of replenishment in coagulation factors that FFP would provide. Further understanding of PIR’s role in the inflammatory response is needed to evaluate its impact during resuscitation.

**Applicability of Research to Practice:**

Plasma inclusive resuscitation may be another tool to treat the multifactorial nature of burn shock and burn induced coagulopathy.

